# The Role of GLP‐1 Agonists in Esthetic Medicine: Exploring the Impact of Semaglutide on Body Contouring and Skin Health

**DOI:** 10.1111/jocd.16716

**Published:** 2024-12-08

**Authors:** Diala Haykal, Barbara Hersant, Hugues Cartier, Jean‐Paul Meningaud

**Affiliations:** ^1^ Private Practice, Centre Laser Palaiseau Palaiseau France; ^2^ Department of Plastic and Cosmetic Reconstructive Surgery Henri Mondor Hospital Créteil France; ^3^ Private Practice, Centre Médical Saint Jean Arras France

**Keywords:** aesthetic Medicine, aging face, body image

## Abstract

**Introduction:**

Glucagon‐like peptide‐1 (GLP‐1) receptor agonists, such as semaglutide, have revolutionized the treatment of diabetes and obesity by promoting significant weight loss through incretin effects. However, rapid weight reduction induced by these medications often leads to esthetic challenges, including facial volume loss, skin laxity, and body contour irregularities. This commentary reviews the esthetic consequences of GLP‐1‐induced weight loss and explores solutions for managing these concerns in clinical practice.

**Methods:**

This review synthesizes available literature and clinical observations to identify key esthetic concerns associated with GLP‐1‐induced weight loss. It examines various treatment modalities, including dermal fillers, biostimulatory agents, energy‐based devices, and surgical procedures. The discussion highlights gaps in empirical data, optimal timing for interventions, and the need for personalized, multimodal treatment strategies. Imaging tools and psychological support are also considered as complementary approaches.

**Conclusion:**

As GLP‐1 agonists become a cornerstone in obesity management, their esthetic implications necessitate a proactive response from esthetic practitioners. Effective management of facial volume loss, skin laxity, and body contour challenges requires a combination of injectable treatments, energy‐based devices, and, in some cases, surgical interventions. Future research should focus on understanding the molecular mechanisms of skin and fat changes and developing standardized guidelines for treating this unique patient population. Additionally, a holistic approach that addresses both physical and psychological outcomes is critical to ensuring patient satisfaction and long‐term well‐being.

## 1 Introduction

Glucagon‐like peptide‐1 (GLP‐1) receptor agonists like Semaglutide have transformed the landscape of metabolic treatment for diabetes and obesity. Through their incretin effects, GLP‐1 agonists help regulate blood glucose levels and promote satiety, leading to significant weight loss [1]. However, recent clinical observations have identified a new esthetic challenge associated with the use of these drugs, the rapid and sometimes extreme weight reduction often leaves patients with excess skin, reduced facial volume, and an overall appearance of premature aging [2, 3]. These esthetic concerns have added complexity to the patient experience, as the physical changes do not always align with the healthier lifestyle outcomes that many expect. In recent years, the role of social media has further amplified the popularity and visibility of GLP‐1 agonists like Semaglutide, with platforms such as Instagram, TikTok, and Twitter filled with testimonials, weight loss transformations, and discussions about “Semaglutide face” [4]. The viral nature of this content has not only raised public awareness but has also contributed to making Semaglutide a trending topic in both the medical and esthetic fields. While many users celebrate the dramatic weight loss results, the widespread attention has also brought to light the esthetic consequences of rapid fat loss, leading to an increasing demand for solutions to counteract these effects [5].

This commentary reviews the esthetic consequences of weight loss induced by Semaglutide, addressing both the challenges and the opportunities for esthetic practitioners in managing body contour and skin quality changes in this population.

## 2 Mechanism of GLP‐1 Agonists

Semaglutide, as a GLP‐1 receptor agonist, enhances glucose‐dependent insulin secretion, suppresses glucagon secretion, and delays gastric emptying. These mechanisms make it an effective tool for controlling blood sugar levels in patients with diabetes. In addition, it significantly impacts body weight by promoting satiety and reducing food intake, through mechanisms that lead to dramatic weight loss in patients with obesity. While the clinical effects of GLP‐1 agonists in metabolic health are well established, their esthetic effects, particularly in the context of fat redistribution and skin integrity lack expansive research [6, 7, 8].

## 3 Esthetic Challenges Associated With Semaglutide‐Induced Weight Loss

### 3.1 Facial Aging and Volume Loss

One of the most striking effects of rapid weight loss due to GLP‐1 agonists like Semaglutide is the significant reduction in facial fat, particularly in the subcutaneous layers, commonly referred to as “Semaglutide face.” The face loses its natural volume, leading to a hollow or gaunt appearance, especially around the midface, temples, and periorbital areas. This can create an imbalance in facial harmony, as the fat pads that normally provide youthful contour support begin to diminish. The loss of these fat pads exacerbates sagging skin, particularly along the jawline and around the mouth, leading to jowling and a less‐defined facial structure [3, 4]. This volume loss often results in patients appearing older than their biological age, creating a psychological impact that can be disconcerting, especially for those who have worked hard to achieve their weight loss goals [9]. The gaunt appearance, associated with the Semaglutide face, contrasts with the expected benefits of feeling healthier, leaving patients concerned about their facial esthetics [10]. To address these concerns, practitioners often use dermal fillers, which can restore lost volume by replenishing deep fat compartments and providing structural support. Biostimulatory fillers, such as calcium hydroxylapatite (CaHA) and poly‐L‐lactic acid (PLLA), offer the additional benefit of stimulating collagen production, which can help rebuild the skin's underlying support structures over time, further improving facial contours. Moreover, fat grafting or volumizing treatments can be employed to target areas where volume loss is most apparent, offering a more natural rejuvenation by reintroducing fat into depleted regions of the face. As Semaglutide continues to be used in weight loss treatments, esthetic providers need to be aware of the potential for significant facial aging and be prepared to provide personalized treatments that can restore the natural balance of facial proportions.

### 3.2 Skin Laxity

Skin laxity is another major esthetic concern for patients who experience rapid weight loss with Semaglutide. The skin's ability to retract and conform to a new body shape is limited by factors such as age, genetics, and the rate of weight loss. When weight is lost quickly, the skin does not have time to adjust, leading to sagging areas that are particularly noticeable in the face, neck, arms, abdomen, and thighs. The loss of the fat that once supported the skin exacerbates this issue, resulting in folds and wrinkles that can detract from the overall benefits of weight reduction. In the face, skin laxity often manifests as drooping cheeks, deepened nasolabial folds, and neck sagging, which further accentuate the appearance of aging. For the body, patients may experience skin folds that hinder mobility or cause discomfort, particularly in the arms, thighs, and abdominal region. This excess skin can lead to functional challenges as well as esthetic concerns, making it difficult for patients to enjoy the full benefits of their weight loss journey [11]. Nonsurgical skin tightening treatments, such as radiofrequency (RF), high‐intensity focused ultrasound (HIFU) or fractional lasers can help stimulate collagen and elastin production to improve skin firmness and elasticity. These treatments are less invasive options for patients who are not candidates for surgery or prefer a more gradual improvement in skin laxity.

### 3.3 Body Contouring

Postweight loss patients, especially those treated with Semaglutide, frequently seek body contouring procedures to refine their body shape and eliminate excess skin. The significant weight loss induced by GLP‐1 agonists often results in areas of the body where skin and tissues do not conform to the new contours, leading to an esthetically displeasing or uncomfortable excess. Body contouring has become an essential part of the postweight loss transformation, which helps enhance the patient's overall body profile and ensures that they can fully enjoy the results of their weight loss. Noninvasive body contouring options, such as cryolipolysis, HIFU, or RF‐based treatments, provide alternatives to surgery for patients with mild–to‐moderate excess skin or fat deposits. These treatments work by breaking down fat cells or stimulating collagen production to tighten and firm the skin. Although effective, they are often used in combination with skin‐tightening modalities to optimize results. These combined approaches can offer significant improvements in body contour without the need for surgery, providing patients with a more streamlined and toned appearance over multiple treatment sessions. For patients with more severe skin laxity issues or large amounts of excess skin, surgical options like abdominoplasty, body lifts, or thigh lifts are often necessary to achieve the desired results. These procedures remove excess skin and tighten the underlying tissues, allowing for a more dramatic and immediate improvement in body shape. Liposuction may also be incorporated to target stubborn fat deposits that remain after weight loss. In many cases, patients benefit from a multimodal approach, where noninvasive treatments are combined with surgical procedures to achieve comprehensive body contouring and long‐lasting results.

## 4 Addressing Esthetic Concerns With Esthetic Interventions

The increasing use of GLP‐1 agonists like Semaglutide for weight management has highlighted the need for esthetic treatments that address the cosmetic challenges associated with rapid weight loss, such as facial volume loss, skin laxity, and changes in body contour. Esthetic practitioners must be prepared to manage these new challenges by offering personalized treatments tailored to each patient's unique needs.

### 4.1 Dermal Fillers and Volume Restoration

The use of dermal fillers, particularly hyaluronic acid (HA) fillers, has become a cornerstone in the treatment of facial volume loss associated with rapid weight loss from GLP‐1 agonists like Semaglutide. HA fillers are highly effective in replenishing lost volume and restoring facial contours, especially in areas, such as the midface, jawline, and temples, where volume depletion is most pronounced. This is crucial because the midface plays a central role in maintaining a youthful appearance, and fat loss in this region often leads to deepened nasolabial folds and a hollowed look under the eyes. HA fillers can restore the plumpness of these areas, creating a more lifted and rejuvenated appearance [12].

In addition to HA, biostimulatory fillers like CaHA and PLLA offer long‐term benefits by stimulating collagen production. These agents provide immediate volumization and promote tissue remodeling over time. CaHA, for instance, can add structural support, particularly along the jawline and cheekbones, enhancing facial definition. PLLA works more gradually, encouraging the body to produce collagen, which leads to firmer, more youthful‐looking skin over several months. These fillers are ideal for patients looking for both immediate and sustained improvements in facial volume and contour. By combining volume restoration with natural collagen stimulation, dermal fillers can help achieve a more harmonious and long‐lasting rejuvenation.

### 4.2 Skin Tightening Treatments

Nonsurgical skin tightening treatments have become increasingly popular among patients experiencing mild–to‐moderate skin laxity following significant weight loss. Technologies, such as RF, ultrasound, and fractional lasers, have proven to be effective in stimulating collagen production, which helps to firm and tighten sagging skin. RF‐based treatments work by delivering controlled heat to the dermis, encouraging collagen remodeling and contraction over time. This makes RF particularly suitable for improving skin laxity in areas like the lower face, neck, and décolleté, where patients commonly notice sagging postweight loss. Ultrasound technologies, such as HIFU, target deeper layers of the skin and underlying tissues to promote lifting and tightening without the need for invasive procedures. These treatments are ideal for patients who are not yet candidates for surgical lifts but want to address early signs of sagging and laxity. These technologies are particularly beneficial for patients with moderate laxity who desire noticeable improvements without undergoing surgery. Even for patients with more advanced skin laxity who still prefer nonsurgical options, fractional radiofrequency microneedling devices offer a compelling solution. By creating microinjuries in the skin, these devices stimulate a natural healing response, boosting collagen and elastin production [13]. This improves skin texture, firmness, and elasticity, making it an excellent option for enhancing the skin's overall appearance and resilience.

### 4.3 Surgical Interventions

For patients with significant skin laxity, especially in areas like the lower face, neck, and body, noninvasive treatments, may not provide sufficient improvement. In such cases, surgical procedures, such as facelifts, neck lifts, and abdominoplasty, become necessary to achieve more dramatic results [14]. These procedures offer long‐lasting outcomes for patients who experience severe sagging after weight loss, particularly in cases where nonsurgical methods cannot fully address the degree of laxity. Furthermore, surgical interventions can be enhanced in combination with volumizing treatments, such as fat grafting or dermal filler injections. Fat grafting involves harvesting fat from other areas of the body and reinjecting it into facial regions that have experienced volume loss [15]. This dual approach allows for the restoration of both skin tightness and facial volume, offering comprehensive rejuvenation. Additionally, combining these surgical interventions with noninvasive skin tightening treatments can optimize esthetic outcomes, restoring a youthful and toned appearance while minimizing scarring and recovery time.

### 4.4 Combination Therapies

The growing trend toward combination therapies has yielded promising results in addressing the esthetic concerns of postweight loss patients. Combining noninvasive skin‐tightening technologies with injectable treatments like dermal fillers, practitioners can achieve more comprehensive facial rejuvenation. The synergistic effects of energy‐based devices (EBDs), such as RF and ultrasound, along with fillers, allow for both skin tightening and volume restoration in a single treatment plan. For example, RF treatments can stimulate collagen production to tighten sagging skin, while fillers restore lost volume and contour, particularly in areas like the midface, jawline, and periorbital regions. This multimodal approach is particularly effective in treating facial aging following rapid weight loss, where skin laxity and hollowed features coexist. Treatments can be tailored to specific problem areas, such as using fractional lasers and fillers to address sagging and volume loss in the midface, while RF and HA fillers enhance the jawline and neck. Combination therapies also allow for a more gradual improvement, with patients experiencing both immediate and long‐term benefits [16]. By utilizing multiple modalities, esthetic practitioners can offer more personalized and effective solutions, ensuring a more harmonious and natural‐looking result for postweight loss patients.

## 5 Research Gaps and Future Directions

The rise in the use of GLP‐1 agonists like Semaglutide for obesity management has created new challenges for esthetic practitioners, particularly in addressing skin laxity, fat redistribution, and changes in body contour following significant weight loss. Current treatments, such as dermal fillers and skin‐tightening technologies, offer solutions, but there is a pressing need for further research to understand the long‐term esthetic effects of GLP‐1 agonists. Rapid weight loss has been shown to impair skin's ability to regenerate and maintain elasticity, leading to sagging and wrinkles, especially in areas with high‐fat content. A deeper understanding of how weight loss affects collagen, elastin, and the extracellular matrix is essential for developing more effective esthetic treatments [17, 18].

### 5.1 Lack of Empirical Data

Despite growing clinical observations, the literature lacks comprehensive empirical data to support some of the esthetic treatments proposed, such as dermal fillers or skin‐tightening technologies. Many of the suggestions discussed are based on practitioner experiences and the rising demand for esthetic interventions among patients undergoing rapid weight loss. To validate these approaches, future research must focus on large‐scale clinical trials and prospective studies that rigorously assess the efficacy of these treatments. By expanding the research base, we can establish standardized guidelines for managing issues like “Semaglutide face” and similar conditions [19, 20].

### 5.2 Variable Impact on Fat Compartments

A significant question for researchers is why certain fat compartments, particularly superficial ones, are more affected by weight loss than deeper compartments. Is this due to a direct action of GLP‐1 agonists on fat cells, or is it the result of the rapid and substantial weight loss itself? Understanding the variability in fat melting across different body regions will require more investigation into the underlying mechanisms and tissue‐specific responses to GLP‐1 agonists.

### 5.3 Potential Targets: Muscles, Ligaments, and Skin

Beyond fat loss, it is critical to understand how these medications affect other structural components, including muscles, ligaments, and skin. The impact on muscle tone and skin retraction is not yet fully understood, particularly in the context of esthetic interventions. A better grasp of how these structures respond to rapid weight loss will help practitioners refine treatment strategies for managing skin laxity and subcutaneous tissue loss.

### 5.4 Timing of Esthetic Interventions

A central challenge is determining the optimal timing for esthetic interventions in patients using GLP‐1 agonists. Should treatments be initiated early during the weight loss process to prevent esthetic issues, or should practitioners wait until weight stabilization, perhaps 3, 6, or 9 months after weight loss? This decision may also depend on the type of products used. For instance, HA fillers, PLLA, biostimulatory agents, and lipofilling have varying effects on tissue stimulation and volume restoration, which could influence the timing and approach to treatment.

### 5.5 Preventive Versus Postweight Loss Treatments

Another key question is whether preventive measures should be taken during the weight loss process, using injectables to minimize fat loss and skin sagging, or whether it is better to wait until the patient's weight has stabilized. The choice between fat restoration and biostimulatory agents would depend on the patient's stage of weight loss and the condition of their tissues.

### 5.6 Imaging Tools: Ultrasound or MRI

Last, using imaging tools such as ultrasound or MRI could be beneficial in assessing the precise impact of rapid weight loss on tissues. These tools would provide valuable insights into fat distribution, tissue integrity, and changes in deeper structures, helping practitioners plan esthetic interventions more effectively. By leveraging advanced imaging techniques, practitioners can tailor treatments to each patient's unique anatomy and needs.

### 5.7 Psychological and Emotional Impact

The psychological toll of rapid weight loss, particularly when patients experience unexpected esthetic changes, should not be underestimated [9]. Many individuals may struggle with the contrast between their expectations of a healthier, more youthful appearance and the reality of skin laxity, volume loss, or an aged appearance. This disconnect can lead to dissatisfaction or even emotional distress, as patients grapple with a self‐image that no longer aligns with how they perceive themselves. It is crucial for esthetic practitioners to address these psychological aspects alongside the physical ones. Research into patient satisfaction, as well as the emotional and mental well‐being of individuals undergoing esthetic treatments postweight loss, is critical. Such research could help practitioners not only restore physical appearance but also support a patient's overall quality of life, ensuring that esthetic interventions contribute positively to both body confidence and mental health. By incorporating a holistic approach that considers both physical and psychological outcomes, practitioners can enhance patient care and long‐term satisfaction.

## 6 Conclusion

As the use of GLP‐1 agonists like Semaglutide becomes increasingly popular for managing obesity, the aesthetic consequences of rapid weight loss are becoming more apparent. While these medications offer significant metabolic benefits, they also introduce new challenges in esthetic medicine, particularly in managing facial volume loss, skin laxity, and body contouring. The accelerated aging effects, such as hollowed features and sagging skin, have prompted a rising demand for personalized esthetic interventions that address these issues. Dermal fillers, biostimulatory agents, and skin‐tightening technologies are playing a pivotal role in helping patients restore lost volume, improve skin elasticity, and refine body contours. However, further research is essential to better understand the long‐term esthetic impact of GLP‐1 agonists like Semaglutide, particularly regarding skin health and fat distribution. Investigating the molecular mechanisms involved in collagen degradation, fat loss, and skin elasticity will allow practitioners to refine and enhance current treatment protocols, offering more effective solutions for postweight loss patients. Moreover, exploring the psychological effects of these esthetic changes will be crucial to providing a holistic approach to patient care. As more individuals turn to Semaglutide for weight loss, the esthetic community must adapt to this growing trend by developing comprehensive and innovative treatments that not only improve physical appearance but also support overall well‐being. In this evolving landscape, the integration of esthetic and medical care will become increasingly important for helping patients achieve both their health and esthetic goals.

## Ethics Statement

The authors declare human ethics approval was not needed for this study.

## Conflicts of Interest

D.H. has received honoraria and research support from L'Oréal, LVMH Research, Vivacy, Galderma, Relife, Fillmed, Merz, Revitacare, Candela, and Hydrafacial. B.H. has received honoraria from Vivacy. H.C. has received honoraria research and clinical trials from Vivacy, Galderma, Evolus, Fillmed, Ipsen, and IMCAS Comexposium health care as a scientific director. J.‐P.M. has received honoraria from Vivacy, Croma, and AIME Academy.

## Data Availability

The data that support the findings of this study are available upon references' part.
